# Trastuzumab with either docetaxel or vinorelbine as first-line treatment for patients with HER2-positive advanced breast cancer: a retrospective comparison

**DOI:** 10.1186/1471-2407-10-28

**Published:** 2010-02-01

**Authors:** Stefania Redana, Michela Donadio, Franco Nolè, Maria Elena Jacomuzzi, Alessandra Beano, Rossella Martinello, Anna Sapino, Giuseppe Viale, Massimo Aglietta, Filippo Montemurro

**Affiliations:** 1 Institute for Cancer Research and Treatment, Division of Medical Oncology, Candiolo Italy; 2 Molinette Hospital, Centro Oncologico Subalpino (COES), Turin, Italy; 3 European Institute of Oncology (IEO), Department of Medical Oncology, Milan, Italy; 4 Mauriziano Hospital, Division of Gynecology, Torino, Italy; 5 University of Turin, Department of Biomedical Sciences and Human Oncology, Turin, Italy; 6 European Institute of Oncology (IEO), Division of Surgical Pathology and Laboratory Medicine, Milan, Italy

## Abstract

**Background:**

Combinations of trastuzumab with either docetaxel or vinorelbine are considered valuable treatment options for HER2-positive metastatic breast cancer patients. We performed a retrospective comparison of the clinical outcomes associated with either one of these combinations.

**Methods:**

From a multi-institutional database we retrieved 179 patients treated with either docetaxel or vinorelbine plus trastuzumab as first-line therapy for HER2-positive advanced breast cancer.

**Results:**

Docetaxel-trastuzumab was superior to vinorelbine-trastuzumab in terms of response rate (RR: 77 vs 57%, p = 0.01) and median overall survival (OS: 35 vs 23 months, p = 0.04), but not in median time to progression (TTP: 12 vs 10 months, p = 0.53). At multivariate analysis, type of treatment was not associated with TTP but was an independent predictor of OS, with a significant reduction in the risk of death in favor of docetaxel-trastuzumab (HR 0.474, 95% IC 0,303-0.742, p < 0.01).

**Conclusion:**

Docetaxel or vinorelbine, when combined with trastuzumab, provide excellent rates of tumor control in patients with previously untreated HER2-positive advanced breast cancer. Docetaxel may offer some advantage in terms of response rate and resulted in a significantly prolonged overall survival, which, because of the retrospective design of our study, deserves further investigation in prospective trials.

## Background

Trastuzumab, a monoclonal antibody directed against HER2, the product of the c-erbB2 proto-oncogene, represents a major step forward in the treatment of the subset of 20 to 30% human breast cancers carrying this genetic abnormality[1-4]. The combination of trastuzumab and chemotherapy resulted in improved clinical outcomes, compared with chemotherapy alone, in patients with HER2-positive advanced breast cancer[1,2]. On account of the pivotal trial[1] and of a subsequent randomized phase II study (M77001)[2], this monoclonal antibody was registered for the treatment of HER2-positive advanced breast cancer patients in combination with the taxanes paclitaxel and docetaxel.

Based on preclinical observations suggesting additivity or even synergism between trastuzumab and other commonly used cytotoxic agents[5,6], several phase II clinical trials have been subsequently conducted testing different associations. Vinorelbine, a vinca-alkaloid derivative, has shown a remarkable clinical activity in anthracycline pre-treated advanced breast cancer patients[7-9]. Preclinical synergism between vinorelbine and trastuzumab was apparently confirmed in the clinic. Response rates of up to 84% response rates were reported when vinorelbine and trastuzumab were used as first-line treatment in appropriately selected HER2-positive advanced breast cancer patients[10,12,13]. For these reasons, together with the favorable toxicity profile of this compound, vinorelbine represents a possible alternative to taxanes in combination with trastuzumab. To date, the choice between a taxane or vinorelbine as a companion for trastuzumab in the first-line treatment of HER2-positive metastatic breast cancer does not rely on data from direct comparisons. A single trial, which failed its target accrual, is available that sought to compare first-line trastuzumab with vinorelbine or with taxane-based therapies[14]. Although it may be reassuring that in the 81 out of the projected 250 patients that could be randomized no significant differences in the main clinical outcomes were observed, this trial can hardly be considered conclusive with respect to its main objectives. Apart from premature closure, another limitation of this trial is that patients in the taxane based arm received heterogeneous paclitaxel and docetaxel-based combinations.

Due to the potential relevance of the issue of the optimal combination of chemotherapy with trastuzumab and the lack of solid evidence from prospective trials, we undertook a retrospective comparison of trastuzumab with either docetaxel or vinorelbine as first-line treatment for HER2-positive advanced breast cancer.

## Patients and methods

Patients for this analysis were selected from a multi-institutional database containing the clinical data of women with HER2-positive breast cancer receiving trastuzumab-based therapy for metastatic disease and treated at 11 different Institutions in Italy, United Kingdom and Hungary. Investigators at each site were asked to provide data for all the consecutive patients who received at least one infusion of trastuzumab for the treatment of metastatic breast cancer, together with their clinical and pathological characteristics, prior treatments for breast cancer and details of the first trastuzumab-based treatment (drugs and doses, best tumor response, date of further progression, and date of death or of last follow-up visit). As of October 31^st ^2008 the database contained clinical data of 441 patients. We queried the database in order to select patients who had received trastuzumab with either docetaxel or vinorelbine as first-line treatment for HER2-positive disease. Patients selected for this analysis could have received endocrine therapy for metastatic disease but not prior chemotherapy other than in the adjuvant or neoadjuvant setting. HER2 positivity was defined as 3+ score by immunohistochemistry (IHC) using the HercepTest. In the case of 2+ score at IHC, the confirmation of HER2 gene amplification by Fluorescence *in situ *hybridization (FISH) was required. All patients had their specimens centrally reviewed for HER2 status by the HercepTest and, when required, by FISH analysis.

For patients with measurable metastatic disease, we asked the investigators to assess and report tumor response according to the WHO criteria[15]. No centralized review of diagnostic imaging was performed to confirm the tumor responses reported by investigators. Response rate was defined as the proportion of patients achieving complete or partial remission (CR+PR). Time to progression (TTP) and overall survival (OS) were calculated by the Kaplan Meier method for the overall dataset of patients (intent-to-treat analysis) from the date of the first administration of trastuzumab to the date of tumor progression or death by any cause (TTP), or to the date of death (OS).

Patients who were alive were censored at the date of the last follow-up contact. Comparisons between patients characteristics were studied by the Chi Square or the Fischer's exact test for dichotomous variables, and by the Mann Whitney U test for continuous variables. Survival curves were compared by the log-rank test. Additionally, the impact of type of treatment (docetaxel or vinorelbine) and other variables on TTP and OS was studied by Cox Proportional Hazards Analysis. Statistical significance was set at p < 0.05. All the analyses were conducted by the SPSS 15.0 statistical package.

Being a retrospective analysis of clinical outcomes, no specific written informed consent was required for this study. However, the process of data collection was conducted in compliance with the Ethical requirements of each of the participating Institutions.

## Results

### Patients and treatments

A total of 178 women with HER2-positive metastatic breast cancer (IHC 3+ or FISH positive) started trastuzumab with either docetaxel (106) or vinorelbine (72) as first-line treatment between September 1999 and June 2008. Main patient characteristics according to type of chemotherapy are summarized in table 1. Significant imbalances in the two groups were found for stage of disease at first diagnosis of breast cancer, with more patients with stage IV in the docetaxel group, for lung and soft tissue involvement, which again were more frequent in the docetaxel group. Prior exposure to taxanes in the adjuvant setting was more frequent in the vinorelbine group. Differences of borderline statistical significance were also found for HER2 status at IHC, which was more frequently 3+ in the vinorelbine group, and for visceral involvement and ≥ 3 metastatic sites, which were more frequent in the docetaxel group.

**Table 1:** Patients characteristics according to type of chemotherapy added to trastuzumab

	Docetaxel (106)	Vinorelbine (72)	P
Median Age, years (range)	55 (33-73)	54 (29-81)	0.47
HER2 Status			0.05
IHC 3+	86 (81%)	66 (92%)	
IHC 2+^1^	20 (19%)	6 (8%)	
ER positive^2^	50 (47%)	32 (44%)	0.63
PgR positive^3^	34 (32%)	22 (31%)	0.64
Initial disease Stage^4^			< 0.01
I	12 (11%)	7 (10%)	
II and III	71 (67%)	58 (80%)	
IV	23 (22%)	6 (8%)	
Prior adjuvant/neoadjuvant chemotherapy	70 (66%)	55 (76%)	0.14
Prior exposure to anthracycline^5^	62 (58%)	50 (69%)	0.14
Prior exposure to taxanes^5^	16 (15%)	31 (43%)	< 0.01
Prior endocrine therapy^6^	43 (40%)	35 (49%)	0.24
Median DFS in months (range)^7^	21 (0-136)	24 (0-146)	0.33
Sites of metastatic disease			
Liver	58 (55%)	33 (46%)	0.24
Lung	43 (41%)	18 (25%)	0.03
Bone	46 (43%)	30 (42%)	0.82
Soft-tissue/nodes	71 (67%)	37 (51%)	0.04
Central nervous system	4 (4%)	3 (4%)	0.90
Visceral involvement (lung + liver + CNS)	82 (77%)	47 (65%)	0.08
Number metastatic sites			0.09
1	31 (29%)	28 (39%)	
2	30 (28%)	25 (35%)	
≥ 3	45 (42%)	19 (26%)	

Docetaxel was always administered every 3-weeks, at doses per-cycle ranging from 75 to 100 mg/m^2^, and continued for 6 to 8 cycles. Patients achieving at least SD continued with trastuzumab alone as maintenance treatment. By converse, vinorelbine, which was administered weekly, usually at the dose of 25 mg/m^2^, was planned to be continued with trastuzumab until tumor progression or unacceptable toxicity. The median number of administered cycles of docetaxel was 6 (range 1-8). The median number of weekly doses of vinorelbine was 38 (range 3-52).

### Analysis of tumor response

A total of 166 patients had measurable metastatic disease and could be evaluated for tumor response. Tumor responses according to type of treatment, as reported by each investigator at each site, are summarized in table 2.

**Table 2:** Tumor response according chemotherapy associated with trastuzumab (166 evaluable patients)^1^

	Docetaxel + Trastuzumab N = 99	95% C.I.	Vinorelbine + Trastuzumab N = 67	95% C.I.	P*
ORR (CR+PR)	76 (77%)	68%-84%	38 (57%)	45%-68%	0.01
CR	16 (16%)		10 (15%)		
PR	60 (61%)		28 (42%)		
SD	18 (18%)		17 (25%)		
PD	5 (5%)		12 (18%)		

CBR (CR+PR+SD ≥ 6 months)	88 (89%)	81%-94%	51 (76%)	65%-85%	0.07

Abbreviations: C.I., confidence interval: RR, response rate: CR, complete remission: PR, partial remission: SD, stable disease: PD, progressing disease: CBR, clinical benefit rate

^1^For 12 patients (7 receiving docetaxel and 5 receiving vinorelbine) tumor response was not available at the time of the analysis

*Chi square test.

The combination of docetaxel and trastuzumab resulted in superior ORR (77 vs 57%, p = 0.01), which was mainly due an increase in the partial remission rate. Twelve and thirteen patients in the docetaxel and vinorelbine group respectively, achieved a disease stabilization that lasted at least 6 months. The resulting clinical benefit rate was 89% and 76% for docetaxel and vinorelbine, respectively (p = 0.07).

### Analysis of disease-free and overall survival

**Figure 1:**
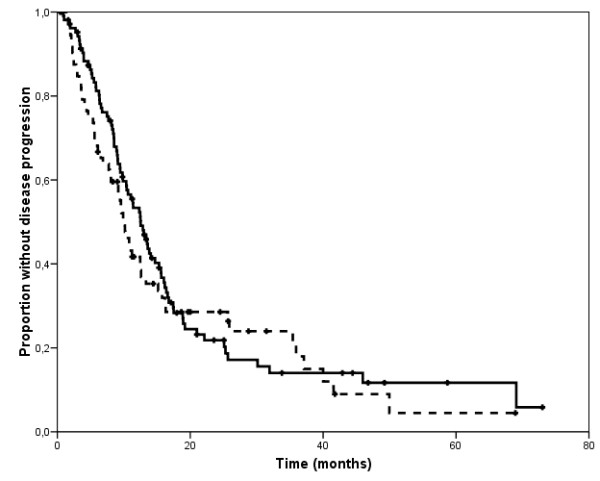
**Kaplan-Meier estimate of time to progression according to type of chemotherapy associated with trastuzumab. The solid line represents patients receiving docetaxel and the dashed line those receiving vinorelbine**.

**Figure 2:**
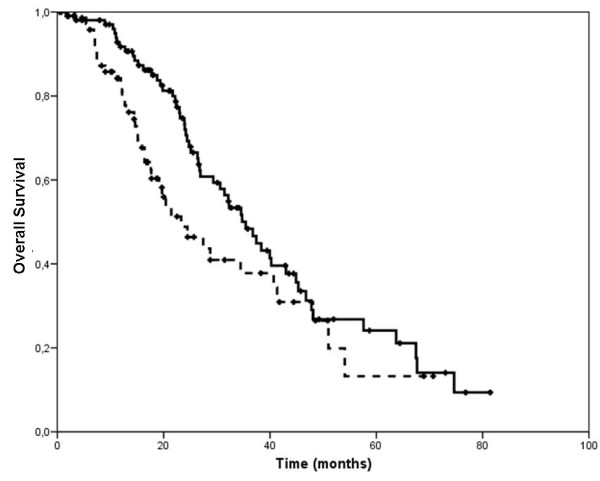
**Kaplan-Meier estimate of overall survival according to type of chemotherapy associated with trastuzumab**. The solid line represents patients receiving docetaxel and the dashed line those receiving vinorelbine.

All 178 patients were included in the survival analyses. The median follow-up for patients who were alive at the time of this analysis was 21 months (range 6-81) and 18 months (range 5-71) for patients receiving trastuzumab with docetaxel or vinorelbine, respectively. In the former group, a total of 80 patients experienced disease progression and 55 died. In the group of patients receiving vinorelbine and trastuzumab, a total of 57 experienced disease progression and 39 died. The Kaplan Meyer estimates of TTP and OS according to type of treatment are displayed in figures 1 and 2. There was no significant difference in median TTP according to treatment type (12 vs 10 months for docetaxel plus trastuzumab and vinorelbine plus trastuzumab, respectively, p = 0.53). Median OS, however, differed significantly in favor of the docetaxel-treated group, with a 12-month gain (35 vs 23 months for docetaxel plus trastuzumab and vinorelbine plus trastuzumab, respectively, p = 0.04).

We then fitted Cox Proportional Regression models of TTP and OS including the following covariates together with type of chemotherapy added to trastuzumab: patient age, which was dichotomized around its overall median value of 55 years, disease-free survival (DFS) from the diagnosis of primary breast cancer to the diagnosis of metastatic disease, which was dichotomized around its overall median value of 21 months, hormone receptor status, HER2 status (3+ vs 2+/FISH positive), receipt of prior adjuvant or neoadjuvant chemotherapy, prior treatment with anthracyclines, prior treatment with taxanes, prior exposure to endocrine therapy, pattern of metastatic disease (liver *vs *other sites), number of metastatic sites (1 vs 2 vs ≥ 3). All the variables, regardless of their significance at univariate analysis, were included into a saturated model and then removed one at a time through a backward elimination selection method using the Wald test at a significance level of p < 0.05. Results of the multivariate analysis are summarized in table 3.

**Table 3:** Multivariate Cox-Proportional Hazard analysis of factors associated with TTP and OS

	TTP			OS		
	
Variable	HR	95% C.I.	P	HR	95% C.I.	P
Type of treatment						
Vinorelbine	1.0			1.0		
Docetaxel	0.765	0.538-1.087	0.14	0.474	0.303-0.742	< 0.01
DFI						
≥ 21 months	1.0			1.0		
< 21 months	1.546	1.092-2.189	0.01	1.945	1.249-3.029	< 0.01
Liver involvement						
No	1.0			1.0		
Yes	1.568	1.1084-2.268	0.02	1.925	1.186-3.125	< 0.01
Number of involved sites						
1	1.0		0.01			
2	1.652	1.071-2.548	0.023	1.568	0.901-2.626	0.12
≥ 3	1.999	1.281-3.119	< 0.01	2.004	1.172-3.427	0.01

TTP, time to progression: OS, overall survival: C.I., confidence interval

Disease-free interval < 21 months, liver involvement and multiple sites of metastatic disease were significantly associated with worse DFS. In the model including these factors plus type of treatment, there was a non significant trend towards reduced risk of progression for patients receiving docetaxel (table 3). The multivariate analysis showed that the same factors predicting TTP were also associated with worse OS and that docetaxel was associated with a statistically significant reduction in the risk of death (table 3).

### Analysis of post-progression treatments and outcomes

**Figure 3:**
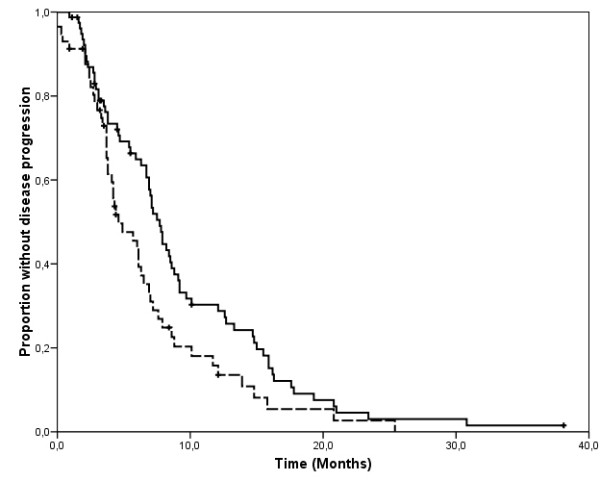
**Kaplan-Meier estimates of time to second progression, measured from the date of second-line therapy initiation to that of tumor progression or death in the absence of progression in patients whose initial treatment was docetaxel plus trastuzumab (solid line) or vinorelbine plus trastuzumab (dashed line)**. The analysis is based on 69 and 49 events occurring in 80 and 57 patients who had initially received trastuzumab with docetaxel or vinorelbine, respectively.

Second-line treatment was known for 124 of 137 patients who progressed during first-line trastuzumab plus either docetaxel or vinorelbine (table 4). Thirty-one patients (38%) crossed over from docetaxel to vinorelbine-based treatments and 17 patients (30%) crossed over from vinorelbine to a taxane. Eighteen patients initially treated with docetaxel and 17 of those treated with vinorelbine continued trastuzumab beyond disease progression in addition to second-line chemotherapy. In a total of 123 patients whose tumor response to second-line therapy was available, the ORR was 24% and 31% for those initially treated with trastuzumab with docetaxel or vinorelbine, respectively (p = 0.51). Median time to progression during salvage chemotherapy was 7.7 months and 4.6 months for patients initially treated with trastuzumab with docetaxel and vinorelbine, respectively (p = 0.02, figure 3).

**Table 4:** Summary of second-line treatments

	Initial Docetaxel + T	Initial Vinorelbine + T
	
Treatment	N 80 (T)^1^	N 57 (T)^1^
Vinorelbine-based	31 (15)	3 (1)
Paclitaxel-based	1 (0)	11 (7)
Docetaxel-based	3 (2)	6 (3)
Capecitabine-based	6 (1)	12 (4)
Trastuzumab^2^	10	6
Anthracycline-based	6 (0)	3 (0)
Endocrine therapy	9 (5)	1 (0)
Miscellaneous	3 (0)	7 (2)
Best supportive care	3 (0)	3 (0)
Treatment not detailed	8	5

^1^Number in parentheses indicate the number of patients who continued trastuzumab beyond disease progression in addition to chemotherapy or endocrine therapy

^2^Patients receiving trastuzumab alone had isolated central nervous system progression and were managed with radiation therapy.

## Discussion

In this retrospective comparison, the combination of docetaxel and trastuzumab was associated with higher response rate and longer overall survival compared with vinorelbine and trastuzumab, with no differences in time to progression. Obviously, because of retrospective nature of the study, several biases need to be taken into account when interpreting our findings. A major source of bias is the fact that the two populations were unbalanced with respect to factors that could have potentially affected clinical outcomes. For example, patients receiving docetaxel had more frequently features of aggressive disease (more patients with stage IV at first diagnosis of breast cancer, with visceral involvement and with ≥ 3 metastatic sites). By converse, patients in the vinorelbine group could be considered relatively chemoresistant because of a more frequent exposure to neo- or adjuvant chemotherapy, and to taxanes in particular. Although we performed multivariate analyses to correct the effect of treatment by these potential covariates, the real impact of these imbalances can not be estimated in this relatively small cohort of patients. Another possible source of bias in our study is the fact that no central revision of tumor response was performed and timing and modality of disease assessment might have been different according to the Institution and the treating physician. In this respect, the higher response rate in the docetaxel-trastuzumab treated group must be considered with caution.

The other relevant finding of our study was the overall survival advantage in the docetaxel group. The endpoint of mortality, which in our database is accurately collected and constantly updated, is much less subject to measurement bias than tumor response and time to progression. We might be conservative and consider also this finding as a possible result of the non-randomized nature of our study.

In spite of that, we believe that this difference in overall survival is worth additional analysis. Studying second-line options and outcomes, we found that time to progression during salvage chemotherapy was significantly longer in patients receiving docetaxel. This possibly implies higher sensitivity to subsequent chemotherapy in these patients, which could be the reason for the improvement in survival. In our opinion, this hypothesis could be true for two reasons, which are not mutually exclusive. The first is that, as already discussed, patients treated with vinorelbine had been more frequently exposed to adjuvant chemotherapy and to taxanes in particular. This exposure may have resulted in increased chemoresistance. The second reason is slightly more speculative. The two regimens that were compared differed in the duration of chemotherapy. All the patients in the docetaxel group received this drug on a 3-weekly basis and for 6-8 cycles, beyond which single agent trastuzumab was administered to non-progressing patients. Conversely weekly vinorelbine was administered with trastuzumab until tumor progression, resulting in longer duration of the combined treatment. If this could have delayed tumor progression, it is also possible that prolonged exposure to vinorelbine could have determined the development of a multidrug resistant phenotype[16], which limited the efficacy of subsequent salvage therapy.

Recently published guidelines indicate both docetaxel and vinorelbine among the preferred first-line agents with trastuzumab despite the absence of randomized comparisons among different combinations[17]. If, on one hand, this implies an assumption of equal efficacy, on the other hand it points at toxicity and convenience as factors to guide therapeutic choices. Unfortunately, we were not able to collect accurate safety data in our study. However, differences between docetaxel and vinorelbine in combination with trastuzumab can be derived from the medical literature and from our previous published experience[2,10,18,19]. Docetaxel is more frequently associated with grade 3 and 4 neutropenia, febrile neutropenia, thrombocytopenia (all grades), alopecia, fluid retention, nail changes and peripheral neuropathy. By converse, vinorelbine, is more frequently associated with constipation and gastrointestinal disorders. Overall, the docetaxel-trastuzumab regimen is, in fact, more toxic than vinorelbine-trastuzumab. Therefore, it is reasonable to prefer vinorelbine and trastuzumab, as many oncologists do, if the assumption of equal efficacy is true. However, we believe that our results, together with those of a randomized study in the adjuvant setting, [20] seem to challenge this assumption.

In high-risk, operable breast cancer patients, docetaxel followed by FEC (5-fluorouracil, epirubicin and cyclophosphamide) outperformed vinorelbine followed by FEC, with a 42% proportional reduction in the hazard of recurrence or death (p = 0.005) [20]. The same study also randomized patients with HER2-positive disease to 9 weeks of trastuzumab given concomitantly with docetaxel or vinorelbine. A recent update of this study confirmed that the addition of trastuzumab to docetaxel, but not to vinorelbine, yielded a significant benefit in terms of time to distant relapse[21].

Based on our findings of an increased overall survival in patients receiving docetaxel-trastuzumab, we believe that the issue of optimal companion cytotoxic agent for trastuzumab in first-line regimens for HER2-positive metastatic breast cancer is worthy of further investigation in adequately designed prospective trials.

## Conclusions

Our retrospective analysis confirms that both docetaxel and vinorelbine, when combined with trastuzumab, provide excellent rates of tumor control in patients with HER2-positive advanced breast cancer. With the increasing number of women receiving docetaxel and trastuzumab as part of adjuvant treatment, our data provide reassurance on combinations of vinorelbine and trastuzumab as first-line option for recurring patients. At the likely price of increased toxicity, docetaxel may offer some advantage in terms of response rate and resulted in a significantly prolonged overall survival, which deserves further investigation.

## Competing interests

The authors of this paper have no potential conflict of interest to disclose.

## Authors' contributions

SR collected clinical data and wrote the manuscript. MD provided clinical data and revised the manuscript. FN provided clinical data and revised the manuscript. MEJ participated in study design and provided clinical data. AB participated in study design and collected clinical data. RM collected clinical data. AS performed centralized FISH analyses on tumor specimens. GV provided clinical data and revised the manuscript. MA revised the manuscript. FM conceived the study, performed statistical analyses, and revised the manuscript.All authors read and approved the final manuscript.

## Pre-publication history

The pre-publication history for this paper can be accessed here:

http://www.biomedcentral.com/1471-2407/10/28/prepub
